# Pediatric Emergency Care Variation: Might Normothetic Data Undermine Adaptive Expertise?

**DOI:** 10.5334/pme.2887

**Published:** 2026-09-30

**Authors:** Laura F. Sartori, Bjorn K. Watsjold, Katherine Wu, Lanelle Quzack, Jonathan S. Ilgen, Joseph J. Zorc, Lara Varpio

**Affiliations:** 1 Division of Emergency Medicine, Children’s Hospital of Philadelphia, Department of Pediatrics, Perelman School of Medicine, University of Pennsylvania, Philadelphia, Pennsylvania, US; 2 Department of Emergency Medicine, University of Washington School of Medicine, Seattle, Washington, US; 3 Children’s Hospital of Philadelphia, Philadelphia, Pennsylvania, US

## Abstract

**Purpose:**

Practice variation across physicians is commonly attributed to idiosyncratic tendencies. What factors contribute to physicians’ practice variation in the disposition of children with asthma exacerbation? How do metrics demonstrating variation impact physicians and learners?

**Methods:**

In this social constructivist qualitative study, we interviewed 11 Pediatric Emergency Medicine attendings, purposively sampling across a spectrum of acute asthma exacerbation admission rates. Participants reflected on their recent care of patients with asthma, how they assess patients, and how they determine disposition. They were shown a de-identified funnel plot demonstrating disposition rates across the physician group and reflected on the 1) quality of the metric and 2) impact on practice. We used reflexive thematic analysis to describe patterns across the data that addressed the study’s research questions.

**Results:**

Participants cited factors at the patient, caregiver, clinical care team, clinical environment, and personal practice levels that might result in variation. Participants framed the ability to weigh these sometimes competing factors as a reflection of patient-centered care. But when presented with a group-level data display, participants were surprised by the variation, valued seeing how they practiced compared to peers, and sometimes expressed a willingness to alter their practices to be “nearer the mean.”

**Conclusions:**

Some variation in care may be driven by physician adaptive expertise, which is not fully captured through metric-driven nomothetic data. Though metrics are fundamental to understanding practice patterns, decontextualized aggregations of data may risk minimizing, or even framing as deviant, the expertise exercised by physicians when they weigh multiple factors in decision-making.

## Introduction

Practice variation in medicine is well documented [1,2,3,4,5,6], and is typically ascribed to physician preferences or idiosyncratic tendencies, rather than systemic differences in the individual patients cared for [2]. Variation in medication management, timing of interventions, and the decision to admit a patient to the hospital all reflect variations in how individual physicians conceptualize risk and anticipate outcomes in the patients they care for [2,7,8,9,10,11].

The ability to weigh a multitude of factors and make timely decisions is key for learners as they gain clinical expertise and for practicing physicians as they refine their care. And yet, our understanding of practice variation is driven by studies of a particular kind. Often, studies on variation leverage large retrospective datasets, which can identify some, but not all, factors associated with variation [12,13,14]. The use of physician “report cards” or other audit and feedback mechanisms then utilize aggregate data to compare physicians to their peers in an attempt to influence practice [15,16,17,18,19]. But these studies lack the nuance to capture individual decision-making behind variation and thus risk framing variation in problematic ways. For instance, such methodologies lack the ability to investigate the multifaceted factors physicians weigh when managing complex patients, how they do or don’t leverage expertise in these scenarios, and the impact of subsequent variation on their clinical confidence. If we want to support learners across the entire medical education continuum to manage—or justify—their practice variations, we need to more specifically examine the factors resulting in variation. Exploring the root causes and the assumptions underlying practice variation provides a first step towards supporting learners as they navigate the multi-faceted, nuanced spaces of medical practice. However, to explore such considerations, different kinds of research methods are required.

We conducted a qualitative study to investigate physicians’ experiences of practice variation. More specifically, we identified a context where this practice variation is experienced, and then set out to answer two more tailored research question: Among Pediatric Emergency Medicine Physicians treating patients with acute asthma exacerbation, what factors contribute to physicians’ practice variation in patient disposition decisions? How do these physicians interpret group level data presentations about practice variation, and how, if at all, might it impact their clinical practice?

To answer these questions, we turned to the care of pediatric asthma exacerbations in the Pediatric Emergency Department (ED). Emergency care requires the ability to make quick decisions regarding patients with a variety of presentations. Research suggests that pediatric emergency medicine (PEM) is a context where considerable practice variation exists with regard to hospital admissions [20,21,22,23,24]. Specifically, asthma exacerbations accounted for over 750,000 ED visits and 70,000 hospitalizations in 2019, and despite specific local and national guidelines for emergent asthma care, there remains variation in management and disposition (i.e. admission vs. discharge home) [25,26,27,28]. Data from the principal investigator’s local PEM context demonstrated substantial variation in asthma admission rates amongst PEM fellowship-trained attendings that persisted even when accounting for patient severity (Figure 1). Such differences in asthma management offer fertile ground to explore the phenomenon of practice variation by elaborating on how individuals working in the same clinical context understand and manage patients with a well-defined and common clinical syndrome. Furthermore, the care of pediatric patients, who are variable in terms of size, vital signs, manifestations of symptoms, and caregiver dynamics, offers a particularly useful context to explore variation.

**Figure 1:**
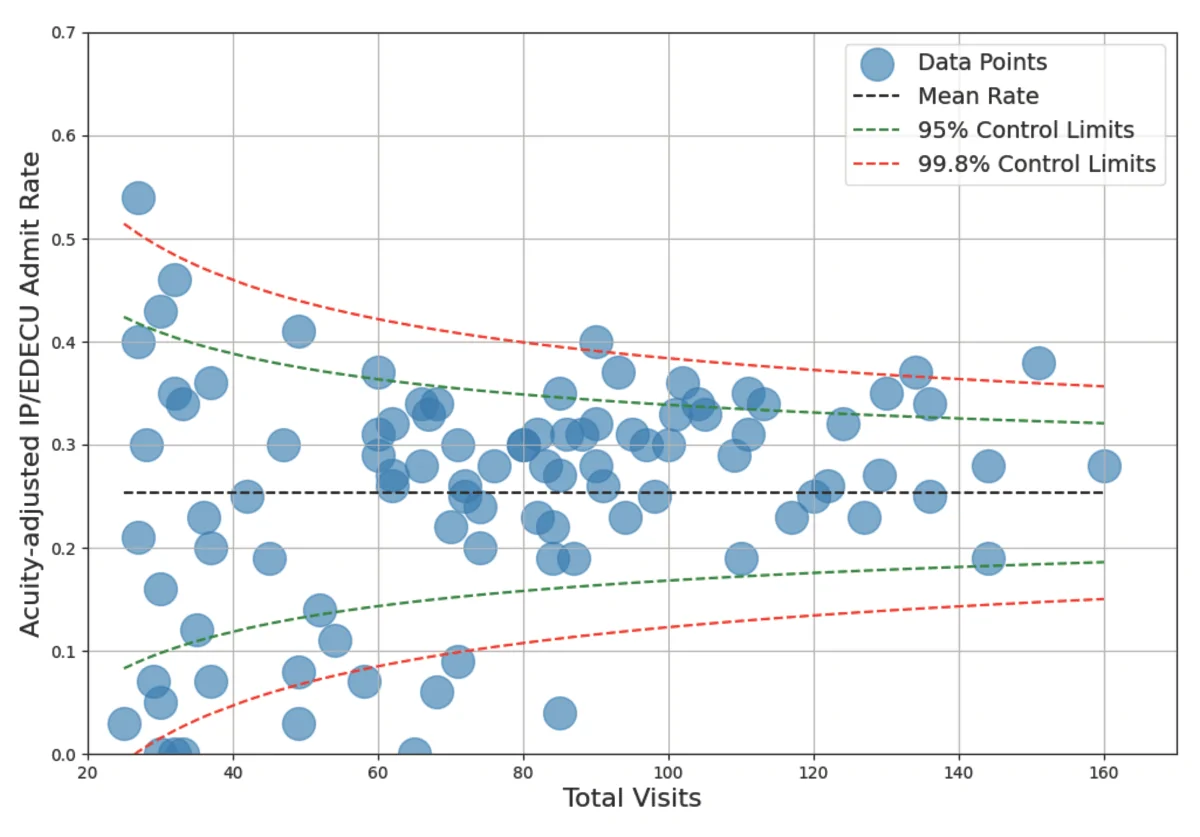
Acuity-Adjusted Emergency Department Inpatient Admission Rate By Attending Physician in Children Presenting with Acute Asthma Exacerbation. Each blue circle represents a Pediatric Emergency Medicine (PEM) Attending Physician, with acuity-adjusted successful discharge rate on the y-axis and number of yearly asthma encounters on the x-axis. The dashed black line represents mean rate among all PEM physicians, with the green dashed line representing 95% control limits and the red dashed line representing 99.8% control limits. Physicians from across the spectrum of discharge rates were selected and interviewed within 1–2 days from last shift in a “respiratory cohort” team in the Emergency Department.

## Methods

This qualitative study was conducted within a social constructivist orientation [29] using reflexive thematic analysis illicited through recollection of specific-patient encounters [30]. The study was submitted to the Children’s Hospital of Philadelphia Institutional Review board and was deemed exempt (IRB #23-021645).

### Participants

We purposively sampled PEM fellowship-trained attendings at a single academic institution using electronic health record-derived data of acuity-adjusted admission rates for children presenting to the ED with acute asthma exacerbation (Figure 1) [31]. Physicians were initially selected based on having at least 20 qualifying patient encounters (defined as a patient presenting to the ED with acute asthma exacerbation) over the past 6 months and classified as high or low admission rate. As the study progressed, additional physicians with admission rates near the statistical average were selected to solicit perspectives across the full spectrum of admission rates. While participants were aware of variations in admission within their practice group from previously-reported data, they were blinded to their own rates within this dataset.

### Data Collection

Between May to November 2024, LS conducted one-on-one interviews with participants. Participants were solicited via email and interviews were performed via Microsoft Teams, 1–2 days after a clinical shift [32]. The 2-part interview guide was developed by LS and LV (see Appendix A for full interview guide). In Part 1, participants were asked to describe two recent cases caring for patients with asthma exacerbations where: 1) care seemed straightforward 2) they felt that their disposition decision-making was challenging. The interviewer probed participants to reflect on specific aspects of the case that influenced their disposition decisions (e.g., to describe moments their care might deviate for expected management, strategies for navigating decisions, and evolution of decisions over time).

In Part 2, participants were shown a funnel plot of admission rates for patients with acute asthma exacerbations specific to their practice group, with anonymized datapoints for each physician (Figure 1). This funnel plot had been presented previously at Divisional meetings and via email, but was not overall well known to participants. Participants were asked to interpret this figure, hypothesize their place in it, and reflect on prior performance feedback. Each interview was recorded, transcribed verbatim, and verified for accuracy against the original recording.

Interviews lasted between 39 and 62 minutes in duration. We determined that sufficient information power had been achieved after conducting 11 interviews thanks to the richness of participant reflections, the relatively narrow focus of our research questions, and our sample specificity [33]. The demographics of these 11 physicians are presented in Table 1; participants had a range of high (n = 4), average (n = 4), and low admission rates (n = 3).

**Table 1:** Participant Characteristics.


SUBJECT	ACADEMIC TITLE	SEX	SHIFTS PER MONTH (Min)	SHIFTS PER MONTH (Max)	YEARS FROM COMPLETION OF PEDIATRIC EMERGENCY MEDICINE FELLOWSHIP	ADMISSION TYPE

01	Assistant Professor	F	8	10	2	Low

02	Associate Professor	F	8	9	10	High

03	Professor	M	5	5	30	High

04	Assistant Professor	F	10	12	3	High

05	Professor	F	8	8	15	High

06	Professor	F	4	4	25	Average

07	Associate Professor	F	11	12	9	Average

08	Assistant Professor	F	11	12	3	Average

09	Professor	F	8	8	25	Low

10	Associate Professor	F	8	9	10	Low

11	Professor	F	6	6	36	Average


### Data Analysis

Six members of the research team (LS, LV, JI, BW, KW, LQ) participated in data analyses. First, LS, KW, and LQ read all the transcripts multiple times to familiarize themselves with the data set; LV, BW, and JI engaged in this process with 4–5 transcripts each. Together, the team developed codes that were iteratively revised as they gained insights into and understanding of physician experiences of practice variability, including the factors contributing to their own decision making and their interpretations of group data presentations. Once the codes had been fully vetted, two investigators (KW, LQ) used NVivo Version 7 (Lumivero, Denver, CO) to code the entire data set. The research team developed initial themes that connected codes to describe patterns across the data that addressed the study’s research questions. As analyses progressed, the team revised the themes to ensure they captured important aspects of the data and to test how different theories could help to inform the final interpretations of the study’s themes (i.e., using the theory-informing inductive data analysis study design) [34].

### Reflexivity

We acknowledge that our individual experiences impacted performance and interpretation of the data. LS is a PEM attending in the same ED as the study participants. Her position in the division could have impacted participant responses, though she is not the direct supervisor of any of the participants. Participants were ensured that their responses were confidential, and great effort was made to affirm their experiences and emphasize that no answer was right or wrong. JI and BW are Emergency Medicine physicians who care for adult patients at a separate institution, which likely shaped their interpretations of participants’ experiences. LV, JI, and BW are educational researchers with expertise in qualitative research. KW and LQ are qualitative experts with no direct clinical experience. The team thus included individuals who practice in ED contexts with experience in practice variability (i.e., an emic perspective) and those not medically-trained, with limited insight into practice variability (i.e., an etic perspective).

## Results

Our results indicate that physicians weigh factors beyond those that might be addressed in traditional clinical pathways, including leveraging past experience, thoughtfully collaborating with team members and families, and carefully accommodating environmental factors. Participants expressed pride in their development of these nuanced skills, providing thoughtful explanations for their disposition decisions. However, when presented with aggregated practice data, participants expressed surprise at the variation presented and a desire to conform to their peers.

### Practice variation is influenced by individual, collaborative, and contextual factors

We identified three themes that captured the broad range of factors that participants attended to when caring for patients with asthma exacerbation. First, participants’ idiosyncratic experiences informed their approaches to case-specific patient factors. Second, participants described a range of team- and family-based factors that shaped their expectant management and monitoring. Finally, participants situated their care of individual patients in the larger context of system- and departmental level influences, detailing how these factors led them to deviate from their “usual” practice.

#### Drawing from experience to alter patient-care decisions

First, we noted that physicians drew significantly from their past experience when making care decisions. For example, participants explained that they had learned over time that asthma, while a common disease with some objective considerations to guide management, could still result in subtle, but crucial, examination findings. As Participant 9 explained, this fact required physicians to think broadly to consider the full array of patient symptoms:

*I think the assessment of patients with asthma is not always straightforward. There are some objective signs, but it’s the constellation of the whole symptoms that make us have a certain decision about them*.

The ability to weigh subtle findings, while comparing past patients with similar presentations, informed participants’ perceptions of an individual patient’s severity, and therefore appropriateness for discharge. For example, multiple participants shared how they *“had learned to fear teenagers with asthma”* (Participant 7), based on instances of rapid, and sometimes unexpected, clinical decompensation in this population.

Participants reviewed their *bouncebacks* (i.e. patients who returned to the ED after being discharged) as a means to regularly learn about the outcomes of the patients they had seen and glean lessons to inform their future practices. Participant 4 described the importance of this practice, stating: *“I always look at my bouncebacks, and if I found that I was having asthma bouncebacks with some frequency, then I would think I’m probably not admitting enough of them.”* Participant 7 noted that the review of bouncebacks changed their steroid prescribing in more mild patients outside of what standard guidelines or peers might suggest:

*I find that sometimes people are like, “oh, they don’t need steroids.” And I’ve just become a steroid sieve because I feel like…all of my bouncebacks are the ones who I didn’t give steroids to*.

#### Leveraging collaborative decision-making to make nuanced decisions

As participants described navigating challenging patient cases, they detailed crucial contributions from family members and members of the healthcare team. They incorporated assessments from others, often over the course of the ED encounter, to support a disposition decision. The participants viewed these discussions not as a challenge to their expertise or lack of confidence, but rather as an indicator of maturity of practice. As Participant 11 described, *“Medicine’s a team sport. I like it when I have people on my team that challenge me and that I know are going to sort of be thinking on their own.”* Care teams (e.g., respiratory therapists, nurses, residents, and other attendings) were also a source of support. Participant 7 described how asking for help from another physician clarified their thought processes: *“Let’s just rehash this history and exam and the trajectory and find out what we’re missing. Is something being pulled over? Are we being hoodwinked?”*

Collaboration was a key strategy utilized, but not all team input was viewed equally, and thus was a source of variation. Participant 8 related how they relied on experienced respiratory therapists who had seen *“thousands of kids with asthma and have seen a lot of different directions.”* Conversly, some team members, including unfamiliar trainees, were seen as potentially less reliable. Participant 5 described *“kind of sizing up who your provider is, because certainly, it takes time to get accomplished at pediatric respiratory assessments.”* Participants related how interpreting their care team members’ assessments was a major contributor to variation in care.

Patients’ families were also sources of variation, providing crucial information that could only be known through shared decision-making. As Participant 3 explained, partnering with families to craft a shared comfort with disposition was a key skill:

*Over time you learn that what we do is really make sure we’re guiding people to the care that we think is best…But you can’t really know what’s going on in that family and why they need to go home, or why that kid wanted to go home*.

Participants assessed and incorporated family comfort, as well as capability, to continue quality care and monitoring outside of the ED. Participant 5 asked specifically about past experiences and comfort with discharge home, relating how a confident family increased their own comfort:

*Some of them are like, “Oh my God. I’ve been dealing with asthma forever. No problem. I will come right back. I know what I’m doing,” and that makes you feel much better*.

These examples reflect how our participants contextualized asthma care as an interaction between the care team in the ED, the patient, and the patient’s family as caregivers outside the hospital.

#### Decision-making is shaped by the broader clinical context

Participants described how in addition to clinical severity, care team, and family considerations, the clinical setting weighed on how they made disposition decisions. Factors such as time of day, hospital and ED capacity, specific location practicing (primary vs. satellite site) and timing of signout to other physicians were all noted to impact disposition decisions.

Participants identified signout between care teams as a pivotal moment in patient care, when they felt an internal pressure to have their care wrapped up. As Participant 4 noted, *“I try whenever possible if I can dispo people before my sign-out.”* At times, this might extend to making dispositions sooner than they typically would. As Participant 8 described,

*If you’re getting towards the end of your shift and you have a kid who is like maybe only an hour and a half out from their treatment but has generally looked reassuring…I might be more inclined to pop in early, do their reassessment early and send them home early*.

Time of day was also noted to be an environmental factor with significant impact on disposition decisions, partly due to the difficult nature of gauging parental comfort and meaningfully engaging in shared decision making. Some noted the overnight hours as a time where they felt particular pressure to make quick disposition decisions for patients, due to fewer resources and longer patient wait times. Subject 6 voiced these pressures,

*In the middle of the night, it’s really hard to tell if the kid is doing better or not. And you have to make faster decisions because you’re the only team there, and there’s always pressure to get kids in and out of the room. So I suspect decisions are made quicker, with maybe a little less data*.

In general, patient volumes in the ED and in the hospital created pressure on participants to advance patient care, including discharging patients who seemed potentially safe but might have benefited from longer observations. Staff shortages also placed constraints on care. At one site, participants described deviating from care pathways when lack of respiratory therapists required that they order only medications that nurses could administer due to hospital policies. In some instances, this led to families recognizing that they could administer similar, or even more appropriate, care at home than was available in the ED. Thus, system-level resources commonly required participants to improvise alternative approaches to disposition.

Overall, participants reflected on a multitude of factors that they felt could cause them to vary in the disposition decisions provided. In general, they reflected on their ability to weigh these factors with a mixture of confidence and humility that they felt was important to providing quality care.

### Interpreting practice metrics: Translating group-level data into individualized implications

In the second part of the interviews, participants were asked to reflect on group level data (Figure 1). Because the data points themselves were de-identified, participants could not reflect on how the data did or did not compare to their previously described perceptions of their care, though they often expressed interest in what their data point might be. Instead, they were asked to reflect on the utility of the data, currently in use at their institution, on their practice. Remarkably, despite having just articulated a plethora of factors shaping disposition decisions, participants voiced a desire to conform to the mean when presented with group-level data. When reviewing the local context’s funnel plot demonstrating variation in individual admission rates, participants’ endorsement of diverse clinical factors supporting variation was seemingly outweighed by their reluctance to be seen as an outlier.

Despite having been presented similar data in past meetings and emails, participants expressed surprise when they saw the variation within the group’s admission practices, uttering expressions of unexpected revelation: *“Wow, that is a lot of variability,”* (Participant 7). Participants valued opportunities to compare their care to their peers, and also carefully examined the plot, hypothesizing about reasons for the variability in the data and their own place in graph, as illustrated by this statement from Participant 8:

*I’m curious where I fall on this and I would want to see where I fall. I also think this plot is completely fascinating because, well, the way I read this plot is it basically doesn’t matter how many qualifying clinical encounters you see, the variation exists regardless*.

In their reflections on the graph, participants’ comments reflected that they considered themselves to be the root cause of the practice variability. For example, Participant 7 suggested that their standing away from the group mean was a reflection of their idiosyncrasies:

*It’d be nice to know if I’m just too conservative and I have to rethink things a little bit or if I’m too loosey-goosey and just discharge people too quickly…If I’m concerned, I worry. I lean more towards the more conservative side*.

Participants pointed to the group mean as a goal to strive for. When presented with anonymous metrics of the whole group, participants frequently expressed a desire to be “average.” As Participant 7 described, *“I want to be perfectly average on like bounce-backs and times to dispo because I don’t need to be an extreme.”* Additionally, some participants expressed a willingness to change their practice if they were one of the outliers. Participant 4 suggested:

*If I am way out of line with other people, then I think, you know, that suggests that maybe there’s something about my practice that should change, whereas if I’m in the ballpark with everybody else I think I would feel really good about that*.

A few participants described the potentially problematic nature of such group metric displays. They reported that metrics and means could cause experienced clinicians to doubt the expertise they had honed over time if they did not fall close to the average. Others feared the impact on early career physicians still working to build confidence. Participant 6 noted:

*I have been on the phone with many of my mentees crying after they’ve gotten their metrics. And I think it makes me worry that people start to think that they have to be towards the mean and not doing what they think is right*.

Finally, some participants expressed distrust of the data and many asked clarifying questions to better understand what variables were included in the graph. They noted that the graph didn’t reflect their professional experiences, and cited differences in types and frequency of shifts worked, as well as patient factors as variables that made them resist the expectation that they should change their practice to better align with the average.

## Discussion

Our research participants easily recalled clinical scenarios in which contextual factors impacted their disposition decisions. Participants described this variation not as a harmful deviation, but as thoughtful and collaborative attentiveness to clinical and environmental factors. However, when presented with an illustration of their group’s aggregate disposition data, participants were surprised by the extent of the practice variability, describing the graph’s mean as the ideal practice pattern, and considered evaluating their personal eccentricities to conform. Only a few participants questioned the desirability of being “average” or the quality of the data presented in the graph. We determined that two theories were particularly applicable and generative: (a) the theory of adaptive expertise and (b) the concepts of idiographic and nomothetic approaches to research.

Our data highlight a noteworthy tension in clinical practice: the expectation that physicians should exercise expertise to make unique patient-centered care decisions vs. the expectation that an individual physician’s decisions should mirror their peers. In other words, there is a contradiction between expectations to be an expert physician and those to be the “average” physician. To understand this tension, we turn to the concept of expertise. Hatano and Inagaki argued that there are two kinds of expertise: *routine expertise* and *adaptive expertise* [35]. Routine expertise involves mastering the knowledge, skills, and attitudes of a domain so that the individual can efficiently perform tasks with high competency. Alternately, adaptive expertise balances such mastery with the capacity to innovate to solve new problems, adapting to challenging situations outside of routine expectations. In medical education, there have been repeated calls for intentionally constructed curricula that support learners in development of adaptive expertise [36,37,38,39].

Applying the concepts of routine and adaptive expertise to explore the findings from our study, we gain new insight into the tension expressed by participants to function both as a highly skilled expert and as an average physician. When faced with straightforward presentations of asthma, physicians should theoretically rely on their routine expertise. They might apply the knowledge, skills, and attitudes they mastered in training to provide excellent patient care. In these situations, we would expect physicians to demonstrate little practice variation. This was demonstrated in reflections on “straightforward” presentations of asthma, such as “clearly sick asthmatics” who received a myriad of asthma treatments and were admitted to the hospital or “clearly well” patients, who presented with routine symptoms, quickly responded to standard care, and were discharged home. In reflections of “less straightforward” patients, our participants related how care was often not routine. Participants incorporated other members of the care team, patients and their families into decision-making. Even among less clinically complex patients, they varied their care to meet the physical and staff resources available to them, responded to hospital and ED-level capacity issues, and changed timing of care decisions to minimize burdens on colleagues. In doing so, they pivoted in ways that did not fit into routine expertise.

Participants described engaging their critical thinking skills to assess the clinical encounter, their team members’ assessments, and family confidence and comfort to flexibly adapt their notions of safe and appropriate disposition to individualized cases. In these situations, instead of a standard and predictable outcome, adaptive expertise *should* create less predictable or uniform outcomes. Though clinicians may see similar proportions of complex situations, by definition, the unique components of those cases result in unique outcomes that routine metrics simply cannot capture.

Therefore, the adaptive expertise framework would suggest that practice variation should be expected or even applauded. We argue that striving to align with the mean should not be the goal for all physicians; instead, the goal should be to nimbly navigate between routine and adaptive expertise in response to patient needs. When interacting with medical learners, physicians should verbalize the thought processes behind their adaptive expertise to not only aid individual decisions, but to normalize variation when practiced with competence. But what does this mean for the data displays that populate physician dashboards? Should we abandon concerns about practice variation? How do we interpret data without unintentionally upholding ideas that conformity in practice is the ideal at all times? For this, we turn to the concepts of ideographic and nomothetic.

Windelband has been credited for defining the terms *ideographic* and *nomothetic* [40]. An ideographic approach to research is concerned with the distinctive qualities of a particular case (e.g. an individual, a clinical department, an organization). An ideographic orientation requires the researcher to analyze and appreciate the unique contexts in which the individual makes meaning and decisions for action. Conversely, a nomothetic approach to research is concerned with articulating the regular patterns that permeate phenomena. A nomothetic approach looks across many different cases to generate law-like statements that can help us predict future outcomes of our decisions and actions. A nomothetic orientation enables the researcher to minimize contextual variation through analysis of large data sets, thereby enabling the researcher to observe the laws that govern the phenomenon.

When we compare the ideographic and nomothetic lenses to interpret our data, we can understand that much of the body of research into practice variation has adopted a nomothetic approach. The funnel plot of admission variation in this study was generated through nomothetic orientation. In contrast, the descriptions of the many factors that shape a physician’s disposition decision to innovatively address unique and complex cases is only visible via ideographic approaches. We do not want to suggest that one or the other of these approaches is “right.” Instead, we suggest that both ideographic and nomothetic studies of practice variation are necessary to generate a nuanced and more complete appreciation for clinical practice. For example, creating an asthma score that includes parental comfort with discharge instructions could balance both metrics (i.e., nomothetic orientations) and the lived environment (i.e., ideographic orientations) in a way that that respects the importance of both these contributions without over-reliance on peer comparisons. Or incorporating feedback mechanisms that use aggregated data to identify trends, but are reviewed with physicians at the specific patient encounter level, to allow appreciation for adaptive expertise. Such considerations are particularly important for junior clinicians and learners, who may require more guidance in understanding and appreciating the role of variation.

### Limitations

Our study was limited to fellowship-trained PEM physician participants, which was intended to highlight experiences of physicians who might practice similarly. The full cohort of clinicians caring for asthmatic children include trainees, advanced practice practitioners, pediatricians, and adult and pediatric emergency medicine physicians. These viewpoints are important and warrant consideration in further studies of expertise development. We examined a single disease-process at a single site in pediatric patients, allowing for deep consideration of disposition decisions that will inevitably vary when applied to other disease processes in other settings. Additionally, families and patients, although important stakeholders in disposition decisions, were not included and should be considered, as clinicians risk mis-interpreting familial factors. Lastly, our examination of disposition is only one type of practice variation. Other types of variation, such as when to provide certain medications, likely vary within the participant group in a different manner than disposition decisions.

## Conclusions

When reflecting on the disposition of patients with asthma, participants described decision-making processes rich in context. They developed strategies for mitigating challenges, and demonstrating adaptive expertise. Yet when faced with clinical metrics comparing them to their peers, participants defaulted to a deference to peer conformity, highlighting a heavy nomothetic influence. We advocate for the development of practice feedback with ideographic consideration for contextual factors and for the normalization of variation for seasoned physicians and learners alike.

## Additional File

The additional file for this article can be found as follows:

10.5334/pme.2887.s1 Appendix A. Interview Guide.

## References

[1] Carden MA, Patil P, Ahmad ME, Lam WA, Joiner CH, Morris CR. Variations in pediatric emergency medicine physician practices for intravenous fluid management in children with sickle cell disease and vaso-occlusive pain: A single institution experience. Pediatr Blood Cancer. 2018;65(1):e26742. DOI: 10.1002/pbc.26742 28766843

[2] Coussens S, Ly DP. Variation in Emergency Department Physician Admitting Practices and Subsequent Mortality. JAMA Intern Med. 2025;185(2):153–160. DOI: 10.1001/jamainternmed.2024.6925 PMC11791704 39714798

[3] Skeith L, Gonsalves C. Identifying the factors influencing practice variation in thrombosis medicine: A qualitative content analysis of published practice-pattern surveys. Thromb Res. 2017;159:52–57. DOI: 10.1016/j.thromres.2017.09.026 28982030

[4] Guterman JJ, Lundberg SR, Scheib GP, et al. Wide Variability in Emergency Physician Admission Rates: A Target to Reduce Costs Without Compromising Quality. West J Emerg Med. 2016;17(5):561–566. DOI: 10.5811/westjem.2016.7.30832 PMC5017840 27625720

[5] Smulowitz PB, O’Malley AJ, Zaborski L, McWilliams JM, Landon BE. Variation In Emergency Department Admission Rates Among Medicare Patients: Does The Physician Matter? Health Aff (Millwood). 2021;40(2):251–257. DOI: 10.1377/hlthaff.2020.00670 33523749

[6] Smulowitz PB, O’Malley AJ, McWilliams JM, Zaborski L, Landon BE. Variation in Rates of Hospital Admission from the Emergency Department Among Medicare Patients at the Regional, Hospital, and Physician Levels. Ann Emerg Med. 2021;78(4):474–483. DOI: 10.1016/j.annemergmed.2021.03.020 34148659

[7] Janke AT, Dalton A, Kocher KE, Vashi AA. Clinician-level variation in admission practices for common conditions in Veteran Affairs emergency departments. Acad Emerg Med Off J Soc Acad Emerg Med. 2025;32(8):874–902. DOI: 10.1111/acem.7002040095423

[8] Abualenain J, Frohna WJ, Shesser R, Ding R, Smith M, Pines JM. Emergency department physician-level and hospital-level variation in admission rates. Ann Emerg Med. 2013;61(6):638–643. DOI: 10.1016/j.annemergmed.2013.01.016 23415741

[9] Khidir H, McWilliams JM, O’Malley AJ, Zaborski L, Landon BE, Smulowitz PB. Analysis of Consistency in Emergency Department Physician Variation in Propensity for Admission Across Patient Sociodemographic Groups. JAMA Netw Open. 2021;4(9):e2125193. DOI: 10.1001/jamanetworkopen.2021.25193 PMC8456378 34546373

[10] Bingham N, Dhall R, Montuori M, Padjen K, Gharibo C, Doan L. Variations in Epidural Steroid Injection Practice Patterns by Pain Medicine Physicians in the United States. Pain Physician. 2022;25(6):E857–E862. 36122269

[11] Hubertus V, Viezens L, Stangenberg M, et al. Practice variations in indication, timing and outcome of Multiple Myeloma patients undergoing surgery for vertebral lesions – results from the European M2Spine study group. J Neurooncol. 2025;174(3):765–777. DOI: 10.1007/s11060-025-05085-y PMC12263761 40512280

[12] Knapp JF, Simon SD, Sharma V. Variation and Trends in ED Use of Radiographs for Asthma, Bronchiolitis, and Croup in Children. Pediatrics. 2013;132(2):245–252. DOI: 10.1542/peds.2012-2830 23878045

[13] Jain S, Elon LK, Johnson BA, Frank G, DeGuzman M. Physician Practice Variation in the Pediatric Emergency Department and Its Impact on Resource Use and Quality of Care: *Pediatr Emerg Care*. 2010;26(12):902–908. DOI: 10.1097/PEC.0b013e3181fe9108 21088636

[14] Florin TA, French B, Zorc JJ, Alpern ER, Shah SS. Variation in Emergency Department Diagnostic Testing and Disposition Outcomes in Pneumonia. Pediatrics. 2013;132(2):237–244. DOI: 10.1542/peds.2013-0179 23878049

[15] Whalen M, Maliszewski B, Gardner H, Smyth S. Audit and Feedback: An Evidence-Based Practice Literature Review of Nursing Report Cards. Worldviews Evid Based Nurs. 2021;18(3):170–179. DOI: 10.1111/wvn.12492 33512082

[16] Alishahi ML, Olson K, Brooks-Russell A, Hoppe J, Runyan C. Provider Reactions to Opioid-Prescribing Report Cards. J Public Health Manag Pract. 2022;28(2):E518. DOI: 10.1097/PHH.0000000000001382 34016911

[17] Jain S, Frank G, McCormick K, Wu B, Johnson BA. Impact of Physician Scorecards on Emergency Department Resource Use, Quality, and Efficiency. Pediatrics. 2015;136(3):e670–e679. DOI: 10.1542/peds.2014-2363 26260722

[18] Chamberlain JM, Simon NJE, Bajaj L, et al. Audit and Feedback on Pediatric Emergency Department Performance Measures: A Stepped-Wedge Trial. Hosp Pediatr. 2025;15(11):901–910. DOI: 10.1542/hpeds.2025-008440 41062095

[19] Stanley R, Lillis KA, Zuspan SJ, et al. Development and implementation of a performance measure tool in an academic pediatric research network. Contemp Clin Trials. 2010;31(5):429–437. DOI: 10.1016/j.cct.2010.05.007 20478406

[20] Bourgeois FT, Monuteaux MC, Stack AM, Neuman MI. Variation in emergency department admission rates in US children’s hospitals. Pediatrics. 2014;134(3):539–545. DOI: 10.1542/peds.2014-1278 PMC4144003 25113291

[21] Mueller EL, Jacob SA, Cochrane AR, Carroll AE, Bennett WE. Variation in hospital admission from the emergency department for children with cancer: A Pediatric Health Information System study. Pediatr Blood Cancer. 2020;67(6):e28140. DOI: 10.1002/pbc.28140 PMC8955607 32275120

[22] Johnson DW, Adair C, Brant R, Holmwood J, Mitchell I. Differences in admission rates of children with bronchiolitis by pediatric and general emergency departments. Pediatrics. 2002;110(4):e49. DOI: 10.1542/peds.110.4.e49 12359822

[23] Chamberlain JM, Teach SJ, Hayes KL, Badolato G, Goyal MK. Practice Pattern Variation in the Care of Children With Acute Asthma. Acad Emerg Med. 2016;23(2):166–170. DOI: 10.1111/acem.12857 26766222

[24] Simmons JM, Biagini Myers JM, Martin LJ, et al. Ohio Pediatric Asthma Repository: Opportunities to Revise Care Practices to Decrease Time to Physiologic Readiness for Discharge. Hosp Pediatr. 2018;8(6):305–313. DOI: 10.1542/hpeds.2017-0085 PMC8237236 29764909

[25] CDC 2019 National Asthma Data. https://www.cdc.gov/asthma/most_recent_national_asthma_data.htm

[26] Children’s Hospital of Philadelphia Emergency Department Clinical Pathway for Evaluation/Treatment of Children with Asthma. https://www.chop.edu/clinical-pathway/asthma-emergent-care-clinical-pathway

[27] Guidelines for the Diagnosis and Management of Asthma 2007 (EPR-3) | NHLBI, NIH. December 3, 2020. Accessed November 10, 2025. https://www.nhlbi.nih.gov/health-topics/guidelines-for-diagnosis-management-of-asthma

[28] 2020 Focused Updates to the Asthma Management Guidelines | NHLBI, NIH. December 3, 2020. Accessed November 10, 2025. https://www.nhlbi.nih.gov/health-topics/asthma-management-guidelines-2020-updates

[29] Gergen KJ. Social Construction in Context. Sage Publications Ltd; 2001. DOI: 10.4135/9781446219645

[30] Braun V, Clarke V, Hayfield N, Davey L, Jenkinson E. Doing Reflexive Thematic Analysis. In: Bager-Charleson S, McBeath A, editors. Supporting Research in Counselling and Psychotherapy : Qualitative, Quantitative, and Mixed Methods Research. Springer International Publishing; 2022. pp. 19–38. DOI: 10.1007/978-3-031-13942-0_2

[31] Patton MQ. Sampling, Qualitative (Purposeful). In: The Blackwell Encyclopedia of Sociology. John Wiley & Sons, Ltd; 2015. DOI: 10.1002/9781405165518.wbeoss012.pub2

[32] Gray L, Wong-Wylie G, Rempel G, Cook K. Expanding Qualitative Research Interviewing Strategies: Zoom Video Communications. Qual Rep. 2020;25(5):1292–1301. DOI: 10.46743/2160-3715/2020.4212

[33] Malterud K, Siersma VD, Guassora AD. Sample Size in Qualitative Interview Studies: Guided by Information Power. Qual Health Res. 2016;26(13):1753–1760. DOI: 10.1177/1049732315617444 26613970

[34] Varpio L, Paradis E, Uijtdehaage S, Young M. The Distinctions Between Theory, Theoretical Framework, and Conceptual Framework. Acad Med J Assoc Am Med Coll. 2020;95(7):989–994. DOI: 10.1097/ACM.0000000000003075 31725464

[35] Hatano G, Inagaki K. Two courses of expertise. In: Child Development and Education in Japan. A series of books in psychology. W H Freeman/Times Books/Henry Holt & Co; 1986. pp. 262–272.

[36] Mylopoulos M, Kulasegaram K, Woods NN. Developing the experts we need: Fostering adaptive expertise through education. J Eval Clin Pract. 2018;24(3):674–677. DOI: 10.1111/jep.12905 29516651

[37] Steinert Y, Irby DM, Dolmans D. Reframing faculty development practice and research through the lens of adaptive expertise. Med Teach. 2021;43(8):865–867. DOI: 10.1080/0142159X.2021.1931081 34078230

[38] Edje L, Price DW. Training Future Family Physicians to Become Master Adaptive Learners. Fam Med. 2021;53(7):559–566. DOI: 10.22454/FamMed.2021.192268 34101818

[39] Woods NN, Mylopoulos M, Nutik M, Freeman R. Defining the specialist generalist: The imperative for adaptive expertise in family medicine. Can Fam Physician Med Fam Can. 2021;67(5):321–322. DOI: 10.46747/cfp.6705321 PMC8115968 33980620

[40] Windelband W. History and natural science. Theory Psychol. 1998;8(1):5–22. DOI: 10.1177/0959354398081001

