# Supplementary material for: Pediatric Emergency Care Variation: Might Normothetic Data Undermine Adaptive Expertise?
Source: Perspect Med Educ. 2026 Sep 30;15(1):1028–38. doi: 10.5334/pme.2887 (PMC13629558; doi:10.5334/pme.2887)
Supplement: Supplementary file 1 [file pme-15-1-2887-s1.pdf]

Introduction:

Thank you for agreeing to participate in this study. As you know, acute asthma exacerbation is a common reason children present to the Pediatric Emergency Department. Despite asthma being a well-studied disease process frequently encountered by clinicians in the Pediatric ED, there is still significant variation in the care of asthma, particularly when it comes to the decision to admit or discharge these patients. For patients of the same initial acuity level, we know that admission rates vary significantly by provider in our group at CHOP. I'm interested in learning more about why, among a well-trained faculty treating a common disease, this variation exists. To do that, I'd like to explore some of your experiences with patients with asthma, both those you see as "straightforward" and those who, for whatever reason, challenged your decision making.

<u>Warm-up:</u>

1. What is your current academic title (Instructor, Assistant Professor, Associate Professor, Professor)?
2. What academic track are you currently on (Clinical Educator, Academic Clinician, Penn Medicine Clinician, Tenure)?
3. How many clinical shifts do you work in a typical month?
4. How many years out are you from completing your initial PEM training?
5. Can you tell me a little bit about yourself and what brought you to work in the CHOP ED?

<u>Setting the framework of straightforward vs. uncertain asthma patients:</u>

1. In the ED, we are often presented with patients whose recommended care is straightforward and likely to be pathway driven. Other times, we encounter patients whose next steps in care are less clear and who therefore challenge us in our medical decision making. We are interested in understanding what your experiences are like – both with those guidelines-driven patients, and those who—for whatever reason—you feel were more challenging.
    a. So I'd like to ask you now to think back to a recent encounter with an asthma patient who, you knew quite quickly, the treatment and disposition would be fairly straightforward. Can you tell me the story of that patient encounter, from first meeting them to the final disposition?
    b. So, in contrast, I'd like to now ask you to think of a patient who did not have that kind of presentation, who—for whatever reason—you were less sure about and who challenged you in your decision to discharge or admit that patient. Can you tell me the story of that patient encounter? Can you start with meeting them and tell me the story through to disposition?

<u>Consequences of uncertainty</u>

2. As you think about these stories, and about other patients with asthma you have encountered that have challenged your disposition decision, what, if anything do you worry about in those more uncertain situations? Are there particular outcomes or consequences you think about?

    a. (If not previously covered) What about for patients that you admit? What do you worry about for them? What do you know about what happens to these patients?

<u>Overcoming uncertainty</u>

3. Again, as you think about these uncertain situations, can you walk me through what, if any strategies you've developed over time to help you navigate, or even mitigate, the uncertainty you've encountered?
    a. Prompts if needed:
        i. Are there other members of the clinical team you rely on in these situations?
        ii. Are there aspects of your encounter with the patient or family that factor into your approach? How, if at all, do you utilize shared decision making.
        iii. Does what is going on in the ED or hospital as a whole ever factor in?
        iv. What about the time of day? The time of your shift? The day of the week? Things going on with you?
4. Follow-up: have you noticed that how you approach uncertainty has changed over time?

<u>Role of Outcomes in Pediatric Asthma Disposition Decisions:</u>

5. Lastly, as we think about care provided in the Emergency Department, we know that patient encounters are unique and that providers themselves are unique. Yet providing good and equitable care, and therefore providing feedback on how we provide that care, is an important part of the process. I'm going to review some types of feedback and I'd love to hear your thoughts on them
    a. 72-hour returns
        i. Can you tell me what you do or don't know about your patients that return to the ED in 72 hours?
        ii. What do you think of this metric? What does it help you learn? What do you like or dislike about it?
    b. Report cards
        i. Can you tell me what, if any report cards you receive on the care you give?
        ii. What do you think of these report cards? What does it help you learn? What do you like or dislike about it?
    c. Funnel plots
        i. My first question is, have you seen this before? If so, in what context?
        ii. Let me walk you through this. On the x-axis is the number of qualifying patient encounters and the y-axis is the admission rate. Each dot on this plot represents a single attending. The yellow line represents the mean for the entire group. This particular plot represents admissions for ESI 1 and 2 asthmatics. As you can see, for similar patients, the admission rate varies greatly
        iii. What do you think looking at this?
        iv. Where do you think you fall?
        v. Where do you think OTHERS think you fall?
    d. "The mean"

i. What do you think about using means to compare your care to the care of others, either in our division, or nationally? Do you think seeing this information changes how you practice? Do you think it should?

e. In reviewing all of this, and reflecting on all the feedback you have access to, what feedback do you feel like you USE to change your practice? Why?

f. What feedback do you ignore? Why?

Summary/Wrap Up:

6. Lastly, as we reflect back on this conversation, we've talked about a lot of different things that impact the care we provide in the ED. Sometimes this may result in deviating from what is recommended in the asthma pathway. What do you think about that? How do you balance what you are doing in the moment from what the pathway might say? Do you think there's tension there?
